# Community-engaged adaptation and pilot testing of a mental health and substance use screening and referral process in HIV pre-exposure prophylaxis (PrEP) care in the Southern USA

**DOI:** 10.1371/journal.pmen.0000148

**Published:** 2025-04-21

**Authors:** Susan Reif, Sarah M. Wilson, Elena Wilson, Haley Cooper, Andy Weinhold, Amy Corneli

**Affiliations:** 1 Center for Health Policy and Inequalities Research, Duke University Global Health Institute, Durham, North Carolina, United States of America; 2 Department of Psychiatry and Behavioral Sciences, Duke University School of Medicine, Durham, North Carolina, United States of America; 3 Durham VA Health Care System, Durham, North Carolina, United States of America; 4 JBS International, North Bethesda, Maryland, United States of America; 5 Department of Population Health Sciences, Duke University School of Medicine, Durham, North Carolina, United States of America; 6 Duke Clinical Research Institute, Duke University School of Medicine, Durham, North Carolina, United States of America; Hamdard University - Islamabad Campus, Pakistan

## Abstract

Mental health and substance use concerns are prevalent in HIV pre-exposure prophylaxis (PrEP)-seeking and priority populations (e.g., same gender loving black men (SGLBM)), often negatively affecting health care outcomes. Identifying individuals who could benefit from access to effective behavioral health treatment remains suboptimal among these populations. We utilized a community-informed process to adapt an evidence-based behavioral health identification and linkage model, Screening Brief Intervention and Referral to Treatment (SBIRT), to include mental health screening and increase cultural responsiveness for use in PrEP care. We piloted the adapted SBIRT model (SBIRT PrEP) in a PrEP clinic in the U.S. South that serves primarily SGLBM. A total of 29 SGLBM participated in the pilot, which involved answering screening questions about anxiety, depression, alcohol, and drug use on an iPad. Participants met with a PrEP navigator to review screening results using a guided model that incorporated participant self-determination and motivational interviewing techniques. SGLBM and staff completed surveys and qualitative interviews that assessed their perceptions of the program. Study findings indicated that the adapted SBIRT PrEP model was acceptable and valued by clinic clients and staff. Survey and interview responses indicated that most clients believed that: 1) substance use and mental health screening was appropriate and important to include in PrEP services; 2) the program was an effective way to address mental health and substance use concerns; and 3) the duration of the screening process was acceptable. A majority of clients reported feeling comfortable answering behavior health screening questions on the iPad and discussing the results with the PrEP navigator. A larger, more rigorous trial is needed to further test the SBIRT PrEP model. Identifying methods to better address behavioral health concerns is critical to enhance PrEP participation and improve quality of life for individuals receiving PrEP.

## Introduction

Pre-exposure prophylaxis (PrEP) is a critical and highly efficacious tool to reduce the spread of HIV and ultimately reach the goal of zero HIV transmission [1]. However, there are a host of barriers that may diminish individuals’ ability and comfort in initiating and sustaining PrEP, including stigma, mental health concerns, substance use, medical mistrust, and misinformation [2,3]. Barriers to PrEP uptake and persistence are particularly acute among minoritized populations [2]. Additionally, behavioral health concerns are prevalent among PrEP-seeking and priority populations, including same gender-loving men (SGLM) [3–5], and are associated with negative outcomes, such as suboptimal medical engagement and medication adherence [3,5,6]. Despite high levels of mental health and substance use issues, engagement in behavioral health treatment services remains low among SGLM [7]. The 2019 National Survey on Drug Use and Health reported that among Lesbian, Gay and Bisexual (LGB) adults, 86.4% of those with substance use disorders and 45.7% with any mental illness were not in treatment [7]. Barriers to behavioral health treatment among LGBTQ+ (Lesbian, Gay, Bisexual, Transgender, Queer/Questioning) people include insurance/financial concerns, social isolation, internal stigma, and a lack of affirming providers[8,9].

PrEP care clinic settings offer an opportunity to detect and address mental health and substance use concerns. PrEP healthcare staff have regular contact with at-risk individuals and have a rapport with patients that can assist in facilitating sensitive conversations. Although screening for mental health and/or substance use concerns is standardized in some clinics that provide PrEP care, many care providers and clinics do not routinely screen for these concerns [3,10]. The Screening, Brief Intervention and Referral to Treatment (SBIRT) model is an evidence-based behavioral health screening tool that has potential benefit for use in PrEP care[11]. SBIRT includes screening for substance use extent and impact, motivational interviewing for those with impactful or hazardous use, and treatment referrals as needed. SBIRT may be particularly useful in PrEP care because it focuses on nonjudgmentally exploring substance use that could negatively affect the individual, which may be more acceptable to PrEP clients who may not see their substance use as an issue that requires treatment.

Studies examining SBIRT among racially minoritized groups or sexual- and gender-diverse populations are very limited; however, formative research has indicated that to enhance the effectiveness of SBIRT in Black communities, the model must accommodate community-specific cultural factors, including beliefs and experiences, and that provider and institutional trust and communication are key factors essential to consider and address in the SBIRT model [12]. Furthermore, SBIRT has previously been expanded to include mental health screening in addition to substance use screening, which may be a critical addition in PrEP care due to higher levels of mental health concerns among SGLM and transgender populations[13–16].

This study examined the feasibility and acceptability of implementing an adapted version of SBIRT in a Southern PrEP clinic that primarily serves clients from racially minoritized groups. A community-engaged process was used to include mental health screening in the SBIRT protocol and to ensure that the protocol was culturally responsive. Developing an effective model for screening, education, and referral for mental health and substance use among PrEP clients is a critical first step in ameliorating the negative effects of these conditions and promoting PrEP adherence. Efforts to increase PrEP uptake and adherence are particularly critical in the Southern US, which has some of the highest PrEP to need ratios in the country as well as having the highest HIV prevalence and diagnosis rates of any region[17]. Barriers to HIV prevention and care in the region include financial concerns, institutional racism, HIV-related stigma, lack of accessible mental health and medical care.

## Materials and methods

### Adapting SBIRT for use in a PrEP clinic in the United States Deep South

We used a modified framework of the ADAPT-ITT model to guide the adaptation and implementation of SBIRT in this study. The ADAPT-ITT model provides guidance for adapting evidence-based interventions and consists of the following eight phases: (1) Assessment (assessing target population needs and potential evidence-based models), (2) Decision (determining the model to adapt or adopt), (3) Adaptation (identifying adaptation strategies), (4) Production (creating adaptation goals and plans), (5) Topical experts (obtaining sufficient technical assistance), (6) Integration (integrating adaptations), (7) Training, and (8) Testing [18]. In Phase 1, we examined the relevant literature to identify an effective model to screen and address behavioral health that could be incorporated into PrEP care. In Phase 2, we selected SBIRT for adaptation due to the strong evidence base for this model and its relative brevity, which would assist in facilitating clinic acceptance and adoption. In Phases 3-5, we used a community engaged process that involved a working group consisting of community representatives who collectively adapted the intervention. Once adapted, we trained PrEP navigators at a PrEP clinic in the U.S. Deep South to implement the adapted SBIRT intervention. The PrEP navigators were SGLBM with PrEP knowledge and experience. Although the study did not focus solely on SGLBM, we expected that a majority of participants would identify as SGLBM due to the demographic composition of the clinic. In Phase 7, PrEP navigators were trained and they piloted the intervention in the PrEP clinic in Phase 8

We followed an established 5-step guide for developing the working group [19]. The first step was to form a core group, which included the study team, PrEP clinic program director, SBIRT consultants, and representatives from community-based organizations (CBOs)/behavioral health organizations already committed to the project. The core group identified (step 2) and contacted (step 3) other potential participants, including other organizational representatives (e.g., mental health and substance use professionals) and SGLBM and transgender individuals with PrEP experience.

An initial meeting (step 4) was held virtually to build community; discuss working group objectives, vision, and structure; and conduct training on SBIRT (facilitated by SBIRT consultants from the National Council for Mental Wellbeing). The 5th step included facilitating three follow-up meetings to adapt SBIRT to best suit the needs of a primarily minority population engaging in PrEP use. These meetings involved review and input on behavioral health measures, the flow of the intervention, and educational materials for inclusion in the SBIRT program. In Phase 6 (integration), the information gleaned from the working group was used to guide development of the final adapted PrEP model. The working group members were compensated for their time.

### Piloting SBIRT in a southern PrEP clinic

We pilot-tested the adapted SBIRT model (named SBIRT PrEP) at the partner PrEP clinic for 5 months (9/1/2022-2/15/2023). The PrEP navigators used a tablet to facilitate SBIRT PrEP via Qualtrics. Individuals who were initiating PrEP or attending a PrEP follow-up visit were approached by the PrEP navigator about pilot participation. This study was approved by the Duke University Medical Center Institutional Review Board (IRB) and was conducted according to principles in the Declaration of Helsinki. All potential participants were provided details about the study and their right to decline along with their rights as a participant. Interested individuals signed a formal virtual study consent. The initial screening measures for the SBIRT PrEP model involved an 8-question brief screening for mental health (PHQ-2 for depression [20] and GAD-2 for anxiety [21]) and substance use (any recent use of alcohol, tobacco, marijuana, or other illicit drugs) (Table 1). Positive scores on depression or substance use prompted additional brief measures on the iPad to identify needs in these areas (PHQ-9 for depression[22], AUDIT-C for alcohol[23], CUDIT-R for marijuana [24], and DAST-10 for other drug use [25]). Standard AUDIT-C and CUDIT-R cut-offs were used identify problematic use[26,27]. No additional anxiety assessment was included to limit the burden of screening questions. Automatically generated scores were compiled for each measure. The PrEP navigator followed a structured educational and motivational interviewing program in Qualtrics based on the client’s behavioral health screening and motivation for change scores, as measured by a standard motivational interviewing readiness scale of 0-10[28]. The program offered the PrEP navigator areas to target for discussion and motivational interviewing with the client and reminders and tips to help guide them through the process. Aspects of the SBIRT Role Play Guideline, which was developed as part of a Substance Abuse and Mental Health Services Administration (SAMHSA)-funded project[29], were adapted for use in the educational and motivational interviewing program. The program was designed to be brief enough (15-20 minutes or less) to be feasible for integration into a clinic visit. If additional time was needed, the PrEP navigator and client would schedule further phone or in-person sessions. Clients interested in mental health or substance use services were referred to available services depending on their needs, preferences, and insurance status. The list of available resources was compiled by the study staff and reviewed by the working group. The program protocol included follow-up contact with the client to determine whether the client received the referred behavioral health services and to address any barriers to linkage or engagement with these services.

**Table 1:** Screening measures.

	Initial screening measure	Secondary screening measure
Depression	PHQ-2	PHQ-9
Anxiety	GAD-2	
Alcohol	1^st^ item of AUDIT-C	Remaining AUDIT-C items
Cannabis	Any use of cannabis	CUDIT-R
Non-cannabis drug use	Any use of drugs (other than Cannabis)	DAST
Tobacco	Any use	

AUDIT-C, Alcohol Use Disorders Identification Test; CUDIT-R, Cannabis Use Disorder Identification Test-Revised; DAST, Drug Abuse Screening Test; GAD-2, Generalized Anxiety Disorder 2-Item; PHQ-2, Patient Health Questionnaire-2; PHQ-9, Patient Health Questionnaire-9.

### Assessment of feasibility and acceptability

We evaluated program feasibility through qualitative, semi-structured interviews (SSIs) with intervention clinic staff. We assessed program acceptability through interviews with intervention staff and structured interviews, surveys, and SSIs with clinic clients who participated in the program.

The study staff conducted structured interviews with clients by phone, generally within 48 hours of the clinic visit. Closed- and open-ended questions focused on client perceptions of the importance/value of screening and their impressions of the SBIRT PrEP screening process, including their comfort with the process, the intrusiveness of the process, and their ability to honestly answer sensitive questions about their behavioral health on the tablet and with the PrEP navigator. Additionally, clients were asked about the usefulness of any related educational materials they received and their perspectives on improving the SBIRT process. One month later, the clinic clients completed an online survey that contained (Table 1), questions adapted from preexisting and tested scales, including the Intervention Acceptability and Tolerability (IAT) scale, Treatment Acceptability and Adherence Scale (TAAS), and Brief Intervention Satisfaction Survey (BISS), which assessed client comfort with the SBIRT process and the acceptability and usefulness of the intervention after time to reflect on the process [30–32]. Clients were asked whether they had received any mental health or substance use services since participating in the SBIRT program and whether they experienced any barriers to services and completed behavioral health screening questions contained in the original screening on the iPad including the PHQ2, HADS2, the first question on the AUDIT and any use of marijuana and other drugs.

One month after completion of SBIRT screening, we also randomly selected clients to participate in a semi-structured interview (SSI) to gain further insight into their impressions and experiences with SBIRT participation; we replaced client participants who did not respond after five contact attempts. At the end of the pilot, we conducted SSIs with clinic staff who participated in the SBIRT program to assess their perceptions of the acceptability of the intervention and the feasibility of implementing the program at their clinic and within PrEP services in general. Clinic staff were asked to identify barriers and facilitators to SBIRT integration and acceptability of integration, including satisfaction, sustainability, and fit for staff and clients. The interview questions were informed by components of the Theoretical Framework of Acceptability (TFA), including affective attitude—how an individual feels about the intervention; burden—the effort required for the intervention; perceived effectiveness; intervention coherence—the extent to which the participant understands the intervention; and self-efficacy [33]. The SSIs also measured staff perceptions of the feasibility of linkage to behavioral health services.

### Data analysis

We used descriptive statistics to summarize survey findings, including the acceptability of the intervention and the utility of educational materials. To assess preliminary effectiveness, bivariate analyses were conducted using the nonparametric McNemar Test [34,35] to assess for changes in mental health and substance use from intake up to the 1-month follow-up survey. McNemar’s Test is a non-parametric test to examine whether there has been a statistically significant change in proportions of a dichotomous variable over time. In this study, it presents a test of the null hypothesis that a scale result on substance use behavior or mental health symptomatology was the same before and after the delivery of SBIRT.

We used adapted rapid qualitative analytic methods to allow for an efficient and timely indexing of themes from the SSIs [36–38]. Each SSI was first summarized by a team member skilled at qualitative data analyses who had familiarity with the subject matter, and the summaries were structurally coded a priori using codes that aligned with research questions contained within the interview guide and expanded upon using open coding during analysis of SSI transcripts. Structural coding reports were reviewed to identify emergent codes, which were applied to interview summaries and main themes were identified through their repetition and emphasis noted in the summaries.

## Results

### Characteristics of the clinic clients

From September 2022 to February 2023, a total of 29 PrEP clinic clients completed the SBIRT process with a PrEP navigator. Of these, 26 participated in the structured interview after SBIRT completion; 24 completed the survey one-month post-participation; and 8 participated in an SSI (5 clients who were selected for an SSI were not able to be contacted). All clients identified as male, and the majority were men of color (Black and/or Latinx) who identified as gay. The average age was 33, and just over half (53.8%, n=14)) had a college degree or higher (Table 2).

**Table 2:** Characteristics of pilot client participants (N=26).

Characteristic	N (%)
**Gender**	
Male	26 (100%)
**Race/Ethnicity**	
Black or AfricanAmerican, Not Hispanic/Latino	17 (63.0%)
Black or African American, Hispanic/Latino	1 (3.7%)
White, Not Hispanic/Latino	3 (11.1%)
White, Hispanic/Latino	2 (7.4%)
Native American, Hispanic/Latino	1 (3.7%)
Other Race/Ethnicity	2 (7.4%)
**Age, yrs**	
18-24	5 (19.2%)
25-34	13 (50.0%)
35-44	6 (23.1%)
45-54	2 (7.7%)
**Education**	
High school diploma/equivalent	3 (11.5%)
Some college	7 (26.9%)
College diploma	12 (46.2%)
Some post-graduate	1 (3.8%)
Post-graduate degree	1 (3.8%)
Vocational/Technical diploma	2 (7.7%)

Nearly one-quarter (24.1%, n=7) of clients screened positive for depression on the PHQ-2 and were directed to complete the PHQ-9. Among those who completed the PHQ-9, 71.4% (17.2% of total sample, n=5) screened positive for a potential depression diagnosis (Table 3). On the brief anxiety scale, 34.4% (n=10) screened positive. For substance use, 89.7% (n=26) used any alcohol, 75.9% (n=22) used alcohol two or more times a month, and 65.5% (n=19) screened positive for potential problematic use on the AUDIT scale. Just over half of the clients (n=15, 51.7%) used marijuana in the last 3 months; 62.1% (n=18) used any tobacco in the last 3 months; and only one individual reported use of other illegal drugs. For clients with any reported cannabis use, 53.3% (n=8) screened positive for problematic use on the CUDIT-R scale. Clients reported an average of 9 out of 10 on the readiness scale to address mental health, 8 of 10 for tobacco use, and 4 for alcohol or drug use. For clients with noted potential problematic alcohol use (n=18), 39% had scores of > 5 (indicating higher motivation) on the readiness to address substance use rating. For problematic cannabis use (n=8), over half (63%) of these clients had a score of >5 on the readiness to address substance use ranking.

**Table 3:** Screening results (N=29).

Mental Health	N(%) or mean(range)
Screened positive for depression (PHQ-2 >2)	7 (24.1%)
Screened positive for anxiety (GAD-2 >2)	10 (34.4%)
Alcohol use (past 3 months)	
Any alcohol use	26 (89.7%)
Alcohol use 2-4 times a month or greater	22 (75.9%)
Positive for potential problematic alcohol use	19 (65.5%)
AUDIT score	4.1 (range: 0-10)
Cannabis use (past 3 months)	
Any cannabis use	15 (51.7%)
Positive for potential problematic cannabis use	8 (27.6%)
Cannabis use daily or almost daily	6 (20.7%)
CUDIT score	4.3 (range 0-26)
Other drug use (past 3 months)	
Any drug use (non-cannabis)	1 (3.4%)
DAST score	0.1 (range 0-3)
Tobacco use (past 3 months)	
Any tobacco use	18 (62.1%)
Daily use of nicotine vape/ vape pen/ Juul/ Puff Bar	5 (17.2%)
Motivation to address mental health (n=10)^	9.2 (range 5-10)
Motivation to address tobacco use (n=11)^#^	8.0 (range 1-10)
Motivation to address alcohol/drug use (n=21)^*^	4.6 (0-10)
Motivation to address alcohol/drug use of greater than 5 on a 19 among those with problematic alcohol use (n=18) Motivation to address alcohol/drug use of greater than 5 on a 19 among those with problematic cannabis use (n=8)	7 (39%) 5 (63%)

^Only asked of individual who screened positive for depression and/or anxiety.

# Only asked of individuals who used any tobacco.

* Only asked of individuals who used any alcohol or drugs.

GAD-2, Generalized Anxiety Disorder 2-Item; PHQ-2, Patient Health Questionnaire-2.

Nearly half of clients (44.8%, n=13) were interested in a referral to behavioral health services, primarily mental health. Of these individuals, 4 connected with behavior health care; 6 were referred but did not connect with services; and 3 were lost to follow-up before they could connect with behavioral health care through the SBIRT program.

### Feasibility and acceptability

#### Post-SBIRT structured interview.

At the baseline interview, a majority of clients believed that the screening was valuable, particularly the mental health screening, as 80.8% (n=21) reported this to be “important” or “very important” and 54% (n=14) reported that screening for substance use was “important” or “very important” (Table 4). Additionally, a majority of clients reported feeling comfortable with the electronic screening process, as 84.6% (n=22) stated feeling “comfortable” or “very comfortable” with answering questions on the tablet regarding substance use, and a slightly higher percentage of clients reported feeling “comfortable” or “very comfortable” with answering the mental health questions on the tablet (n=24, 91.4%). The vast majority of clients reported feeling “comfortable” or “very comfortable” discussing substance use with the PrEP navigator (96.2%, n=25). A majority of clients also reported that they felt “comfortable” or “very comfortable” talking with the navigator about mental health (84.6%, n=22). Several respondents (n=4) described being surprised at being asked the mental health and substance use screening questions and they expressed that more dialogue before engaging in the SBIRT process about content and reassurance about confidentiality would be beneficial. One client suggested that providing assurance that the screening information is gathered to assist the client (i.e., this is *“you looking out for you”*) would improve their comfort level with the process.

**Table 4:** Acceptability measures – directly after SBIRT (n=26).

Concept	N(%)
Importance of screening	
Alcohol and drug use	
Not at all	2 (7.7%)
Somewhat	10 (38.5%)
Important	5 (19.2%)
Very Important	9 (34.6%)
Mental health	
Not at all	2 (7.7%)
Somewhat	3 (11.5%)
Important	6 (23.1%)
Very important	15 (57.7%)
Comfort answering alcohol and drug use questions:	
Tablet	1 (3.8%)
Very uncomfortable	3 (11.5%)
Uncomfortable	11 (42.3%)
Comfortable	11 (42.3%)
Very comfortable	
With PrEP navigator	0 (0%)
Very uncomfortable	1 (3.8%)
Uncomfortable	7 (26.9%)
Comfortable	18 (69.2%)
Very comfortable	
Comfort answering mental health questions:	
Tablet	0 (0%)
Very uncomfortable	2 (7.7%)
Uncomfortable	13 (50.0%)
Comfortable	11 (42.3%)
Very comfortable	
With PrEP navigator	0 (0%)
Very uncomfortable	2 (7.7%)
Uncomfortable	6 (23.1%)
Comfortable	16 (61.5%)
Very comfortable	2 (7.7%)
N/A	
Felt they could be honest about alcohol and drug use	
Tablet	0 (0%)
Not at all	1 (3.8%)
Somewhat	25 (96.2%)
Very	
With PrEP navigator	0 (0%)
Not at all	4 (15.4%)
Somewhat	22 (84.6%)
Very	
Felt they could be honest about mental health	
Tablet	0 (0%)
Not at all	6 (23.1%)
Somewhat	20 (76.9%)
Very	
With PrEP navigator	0 (0%)
Not at all	8 (30.8%)
Somewhat	18 (69.2%)
Very	
Usefulness of information on alcohol and drug use	
Not at all	1 (3.8%)
Slightly useful	4 (15.4%)
Useful	6 (23.1%)
Very useful	5 (19.2%)
Did not discuss	10 (38.5%)
Usefulness of information on mental health	
Not at all	0 (0%)
Slightly useful	2 (7.7%)
Useful	8 (30.8%)
Very useful	6 (23.1%)
Did not discuss	10 (38.5%)

Most clients reported they could be very honest about reporting their substance use and/or mental health, particularly about substance use on the tablet (96.2%, n=25) and with the navigator (84.6%, n=22). Similarly, a greater proportion of clients said they could be “very honest” on the tablet about mental health (76.9%, n=20) compared with the navigator (69.2%, n=18). Client responses to open-ended questions regarding mental health suggested they had greater comfort answering questions honestly on the tablet because this format was not face to face. One person said they would feel more comfortable if they knew the navigator better.

#### Follow-up survey.

The vast majority of clients responded positively about the program (Table 5). For example, clients thought the program would be helpful for those who participated in it (91.7%, n=22) and would be “a good way to learn more about substance use” (83.3%, n=20) and “mental health” (100%, n=24).

**Table 5:** Acceptability measures, one month after SBIRT (n=24).

Item	Strongly Disagree	Disagree	Agree	Strongly Agree	N/A
This screening program will help others who participate in it	2 (8.3%)	0 (0%)	10 (41.7%)	12 (50.0%)	
This screening program is a good way to learn more about mental wellness	0 (0%)	0 (0%)	9 (37.5%)	15 (62.5%)	
This screening program was a good way to learn more about alcohol and drug use	0 (0%)	4 (16.7%)	12 (50.0%)	8 (33.3%)	
This alcohol/drug use screening was acceptable to me	0 (0%)	0 (0%)	14 (58.3%)	10 (41.7%)	
This mental health screening was acceptable to me	1 (4.2%)	0 (0%)	11 (45.8%)	12 (50.0%)	
This screening program provided an effective way to help me address alcohol and/or drug use	0 (0%)	3 (12.5%)	10 (41.7%)	1 (4.2%)	10 (41.7%)
This screening program provided an effective way to help me address mental health	1 (4.2%)	2 (8.3%)	10 (41.7%)	7 (29.2%)	4 (16.7%)
The issue of mental health is important enough to have this screening program	1 (4.2%)	0 (0%)	12 (50.0%)	11 (45.8%)	
The issue of alcohol and drug use is important enough to have this screening program.	1 (4.2%)	0 (0%)	12 (50.0%)	11 (45.8%)	
This screening program helped me to gain useful knowledge about mental health	0 (0%)	2 (8.3%)	14 (58.3%)	8 (33.3%)	
This screening program helped me to gain useful knowledge about alcohol and drug use	0 (0%)	4 (16.7%)	15 (62.5%)	5 (20.8%)	
Overall, I found this screening program to be a waste of time	8 (33.3%)	12 (50.0%)	3 (12.5%)	1 (4.2%)	
Participating in this screening program was distressing to me	12 (50.0%)	8 (33.3%)	3 (12.5%)	1 (4.2%)	
Overall, I found this screening program intrusive	12 (50.0%)	7 (29.2%)	4 (16.7%)	1 (4.2%)	
The amount of time it took to complete the screening program was reasonable	1 (4.2%)	0 (0%)	15 (62.5%)	8 (33.0%)	
The clinic is an appropriate place for conducting the screening program	0 (0%)	1 (4.2%)	13 (54.2%)	10 (41.7%)	
This is an appropriate program to identify alcohol and drug use	0 (0%)	1 (4.2%)	14 (58.3%)	9 (37.5%)	
This is an appropriate program to identify mental health concerns	0 (0%)	3 (12.5%)	13 (54.2%)	8 (33.3%)	
I feel confident that I can continue taking PrEP as prescribed	0 (0%)	0 (0%)	7 (29.2%)	16 (66.7%)	

PrEP, pre-exposure prophylaxis.

All but one client indicated that the mental health screening was “acceptable to me,” and all agreed that the substance use screening was “acceptable to me” (Table 3). Furthermore, a majority of clients agreed or strongly agreed that the program “helped me gain useful knowledge about alcohol and/or drug use” (83.3%, n=20) and “helped me gain useful information about mental health” (91.7%, n=22). A few clients reported that they “found this program to be intrusive” (16.6%, n=5) and reported thinking the “program was a waste of time” (12.5%, n=3).

When asked about the impact of the program on mental health, 70.8% agreed that the program provided an “effective way to help me address mental health;” (12.5%, n=3) disagreed; and 16.7% (n=4) said this was not applicable to their situation. For the same question about alcohol and drug use, 45.8% (n=11) reported that this program was an effective way to address alcohol and drug use; 41.7% (n=10) said this did not apply to their situation; and 12.5% (n=3) disagreed. In terms of the need for a screening program, the vast majority of clients reported that the issue of mental health (95.8%, n=23) and alcohol/drug use (95.8%, n=23) was “important enough to have this screening program.” Most clients (95.8%, n=23) also thought the clinic was an appropriate place to offer the program.

#### Semi-structured interviews (N=8).

Clients described being fairly comfortable discussing their mental health and substance use during their PrEP appointment. Several clients (n=3) emphasized that this was their first experience being asked about mental health at a medical appointment. A couple of interview participants (n=2) emphasized the importance of having the PrEP navigator who they were discussing mental health and substance use issues with to be either a peer or to have some cultural commonalities with the clients. One participant, a Latinx gay male in his early 20s, said of the peer connection:

“*Getting it from someone who’s also in the community helps because it’s like less judgy. I feel more open to discussing that, because I know not a lot of people understand the community…him having that kind of personal level and saying like, yeah, I get it helps me answer the questions more fully and more honestly.”*

Differing cultural norms within the community of young SGLBM concerning discussion of mental health and treatment-seeking behaviors were emphasized by multiple clients (n=3). Additionally, clients noted the normalized use of substances within their community, explaining that while substance use may have been flagged through the SBIRT process, it was often not considered an issue by the individual compared with others in their social network who were engaging more heavily. Thus, some clients did not have high motivation to address substance use as an issue.

Clients varied in their preference for completing the SBIRT process using the tablet to answer questions versus discussion with the PrEP navigator. More clients (n=5) preferred in-person discussion, although notably, concerns about stigma related to substance use and mental health persisted from clients through both delivery methods. Several clients (n=3) suggested approaching the topics of mental health and substance use as intertwined with sexual health, and thus PrEP, to put the discussion within the context of other issues in the clients’ lives. One client, a Black gay male in his mid-20s, provided suggestions for this integration, recommending “*a model that kind of says, hey, you know you come here to talk about your sexual health, but here are other things that also, you know, contribute.”* Clients felt that it was incredibly important to emphasize confidentiality of responses and clear communication about use of the collected data, particularly with regards to technology.

Clients suggested that having more information and more time available in the initial session to discuss referral options would be preferable to coordinating referrals at a later time; a factor that may increase the likelihood of connecting with services.

#### Clinic staff (N=3).

We conducted SSIs with three clinic staff members: a PrEP navigator who delivered SBIRT to clients, the clinic programs director, and an advanced practice provider at the clinic who provided medical care to PrEP clients. Clinic staff described a number of barriers to implementation of SBIRT that were primarily related to clinic structure and function. Staff members reported that screening and referral for mental health and substance use were highly valuable. However, clinic staff described facing challenges in incorporating the screening process as part of clinic workflow. Despite preparatory planning for implementation of SBIRT, staff perceived that more time to identify barriers and facilitators to implementation would have been beneficial. Clinic staff also emphasized challenges with their referral processes, expressing frustration at the inability to determine whether clients attended a referral appointment when a referral was made. They reported desiring more coordinated efforts in community referrals to determine follow-through. They also were interested in increased organizational priority and more widespread staff involvement in implementing SBIRT. They noted that this would have allowed for more coordination and cohesion in addressing positive mental health and substance use screens across the clinical care team within the organization. The limited supply of mental health providers who accept Medicaid or provide free or sliding scale care was another major challenge noted by staff, which complicated their ability to refer clients to services.

#### Research team observations.

The study research team made key observations during the implementation phase of the study. The clinic was challenged during the implementation period with turnover of its two PrEP navigator positions, which disrupted implementation of SBIRT and made it difficult for clients to build consistent rapport with PrEP navigators. Even when the clinic was fully staffed, the lack of staff time and competing priorities limited the ability of team members to make timely mental health and substance use referrals and to follow up with clients on those referrals.

The study team also observed that the inability to integrate the SBIRT protocol into the electronic medical record (EMR) was a challenge. The PrEP navigators were asked to utilize an iPad to complete the SBIRT process while also using the general EMR for medical data, since integrating the two systems was deemed too complex for study scope and timeline. Incorporating the SBIRT program into the EMR would have allowed for more consistent notification to medical staff regarding the SBIRT findings and the needs of clients for intervention and referral.

### Preliminary effectiveness

At the client follow-up interview, 58.3% (n=14) of clients reported alcohol use two or more times a month compared with 91.7% (n=22) at baseline, and 33.3% (n=8) reported any cannabis use compared with 62.5% (n=15) at baseline (see Table 6). For mental health, 17.4% (n=4) of clients screened positive for depression on the PHQ-2 and 26.1% (n=6) screened positive on the GAD-2 brief anxiety scale at follow-up compared with 24.1% (n=7) and 34.5% (n=10) at baseline, respectively. Overall, there were non-significant declines in substance use and mental health-related symptoms. However, these estimates and p-values should be interpreted with caution given that the current study is a pilot and not powered to detect changes over time [39]. One outcome – any cannabis use – showed a marginally significant decline at follow-up (p=0.08).

**Table 6:** Bivariate analysis of mental health and substance use measure (’s Test).

	Baseline (N=29)	One month after SBIRT (N=23)	McNemar ’s Chi-sq and (p-value)
Depression (Positive on PHQ-2)	7 (24.1%)	4 (17.4%)	0.0 (1.0)
Anxiety (Positive on GAD2)	10 (34.5%)	6 (26.1%)	0.33 (0.56)
	**Baseline (N=24)**	**One month after SBIRT(N=24)**	**McNemar’s Chi-sq and (p-value)**
Alcohol (2 to 4 times per month or more)	22 (91.7%)	14 (58.3%)	1.8 (0.18)
Cannabis (Any use)	15 (62.5%)	8 (33.3%)	3.0 (0.08)

Regarding motivation to address behavioral health, over three-quarters of the clients (79.2%, n=19) agreed with the statement that “I am thinking of taking steps to work on my mental wellness;” the remaining 20.8% (n=5) said they did not need to work on mental wellness. For alcohol and drug use, 37.5% (n=9) agreed with the statement “I am thinking of taking steps to work on my alcohol and drug use,” and 58.3% (n=14) reported that they did not need to work on drug and alcohol use. Additionally, relatively high levels of PrEP medication regimen adherence were reported, as 78.3% (n=18) reported taking PrEP as prescribed “all of the time;” 17.4% (n=4) said they take medications as prescribed “most of the time;” and one person stated that they take PrEP “a little of the time.”

## Discussion

Through a community-informed process, SBIRT was adapted to suit implementation at a PrEP clinic primarily serving SGLBM in the U.S. Deep South. Findings from this feasibility pilot study indicated that the services were possible to implement at the clinic and were acceptable and valued by most clients. Client interview and survey data indicated that most clients believed that behavioral health screening was appropriate and important to include in PrEP services. At the interview immediately following screening, clients reported a lower level of importance for alcohol and drug use screening in PrEP services than for mental health screening; however, at the one-month follow-up, this difference had dissipated, and the vast majority of clients reported that mental health and alcohol and drug use were important to screen for in a PrEP program. It is unclear whether this change was influenced by a slight difference in the wording of the question or if, after reflection, clients had increased their perception of the value of alcohol and drug screening. A majority of clients reported feeling comfortable answering behavior health screening questions on the iPad and then later discussing the results with the PrEP coordinator. In addition, most clients who completed the SBIRT PrEP process rated the length of the screening and education as appropriate, although some reported that they would have preferred additional time with the PrEP navigator who facilitated the screening.

Consistent with other research[40], mental health and substance use concerns were detected in a substantial proportion of clients, as nearly one-quarter screened for depressive symptoms and just over one-third for anxiety. For substance use, just over two-thirds screened positive for potential problematic alcohol use and one-quarter for potential problematic cannabis use. Most clients self-reported being highly motivated to address mental health on a readiness rating scale (9 out of 10 on the rating scale). In contrast, readiness to address substance use was much lower (4 out of 10 on the rating scale). This finding may reflect in part, that many of the clients (75% for cannabis and 34% for alcohol use), did not screen positive for problematic use. However, examination of motivation ratings for clients with probably problematic alcohol use, less than half had motivation ratings over five out of 10. Motivation for clients with problematic cannabis use was higher at 63%. For individuals screening positive for potential problematic use, education to enhance self-awareness and offer information about potential behavioral health concerns is needed with the caveat that PrEP clients often may not want to make significant changes in alcohol and drug use. In these cases, harm reduction education tailored to the participant’s stage of readiness is beneficial and was deemed acceptable by clients in this pilot. Additionally, delivery of the SBIRT process by a peer or someone in the clinic with some intersection with the clients was emphasized by clients as decreasing stigma related to substance use and mental health and increasing their comfort addressing these topics within a cultural context. The screening process resulted in counseling referrals for nearly half of clients, primarily for mental health counseling rather than substance use. Just under one-third (31%) of clients were eventually connected with mental health services.

Although the study findings indicated that the SBIRT PrEP intervention was feasible to administer and was reported as acceptable and useful by the vast majority of clients and clinic staff, several important lessons will be critical to address for future implementation and more rigorous evaluation of the program. These factors reflect observations from study staff, clients, and clinic staff:

1) Clinic preparation: Although the research staff collaborated with the PrEP clinic to determine how the SBIRT pilot would be integrated into the clinic, more intense preparations were needed to address staff turnover and competing needs within the clinic. Furthermore, integration of SBIRT PrEP findings and recommendations into the clinic EMR would have been beneficial to simplify the process and assure access to the information for clinic providers.
2) PrEP navigator effort: Sustainment of SBIRT implementation likely relies on ensuring that adequate time is available for the PrEP navigator to facilitate referrals for behavioral health services, follow up with clients on referrals, and assist clients with any identified treatment barriers. Assistance with behavioral health care referrals and care engagement could also be provided by a community partner or contract organization if clinic staff effort is not available.
3) Availability of mental health and substance use resources: Due to behavioral health care provider shortages, it is essential that the clinic and study staff identify community resources for behavioral health to ensure that referrals can be made for care. This is especially true for clients who are uninsured or underinsured, as resources for these individuals are even more limited. If clients are referred and then placed on waiting lists, PrEP delivery organizations need to be aware that behavioral health needs have not yet been met and ongoing monitoring and assistance can be provided as needed.

Additional limitations to the pilot study must be considered when interpreting the results. The sample was relatively small; although the sample size was adequate for an early pilot study, a larger study with multiple clinics would be needed to more rigorously examine the impact of the implementation strategy, including the influence of screening on behavioral health outcomes. In addition, the population was primarily same gender–loving Black cisgender men under age 35; therefore, the findings may not be generalizable to other populations, such as older individuals, cisgender women, and transgender and non-binary individuals.

Despite the study limitations, this pilot study suggests that using SBIRT to incorporate mental health screening into PrEP care is both feasible and acceptable. Furthermore, findings suggest that integration of SBIRT practices in the clinic is critical as is ensuring that adequate peer navigator time is available to refer individuals to care that is available and affordable. Identifying methods to better identify and address behavioral health concerns is vital to enhance PrEP participation and improve quality of life for individuals receiving PrEP.
